# Knowledge, Attitudes, and Practices About the Use of Silver Diamine Fluoride Among Dentists in Riyadh, Saudi Arabia: A Cross-Sectional Study

**DOI:** 10.7759/cureus.60245

**Published:** 2024-05-13

**Authors:** Aljohara Al-Hussyeen, Reem J Alghamdi, Razan S Aljarboua, Rawan A Alayoub, Shoug M Alrashedi

**Affiliations:** 1 Department of Pediatric Dentistry, King Saud University, Riyadh, SAU; 2 Department of Dentistry, King Saud University, Riyadh, SAU

**Keywords:** dental caries, knowledge, practices, attitudes, silver diamine fluoride, pediatric dentists

## Abstract

Introduction

Dental caries is a worldwide disease affecting children and older populations. There are multiple interventions to treat dental caries that could be sometimes hard to deliver, due to the general status of patients such as insufficient cooperation or medically compromising conditions. Therefore, another alternative to control dental caries is being used and has been recently introduced and approved by the Saudi FDA which is silver diamine fluoride (SDF).

Objectives

This study aimed to investigate the level of knowledge, attitudes, and practices about the use of SDF among dentists in Riyadh, Saudi Arabia.

Materials and methods

This cross-sectional study was conducted using pretested, an online digitally designed questionnaire sent to 500 Saudi Commission for Health Specialties (SCFHS) registered dentists working in the following specialties: general practitioners, interns, restorative, family, pediatric, and public health dentists who are working in public and private hospitals and clinics. One-way ANOVA was used to determine the comparison of participants’ knowledge and Practice with three variables (participants’ specialty, place of work, and years of experience), while Chi-square was used for the comparison of respondent’s attitudes about the use of SDF and the above same variables.

Result

A total of 265 responses were received (response rate is 53%). The majority of the respondents were general practitioners and interns 144 (54.3%). Over 50% of dentists believed that they were very informed about SDF. The mean score of knowledge and practices of SDF of participants was found below average 49.13 (19.81) and 43 (26.12), respectively. Correlation of the mean score knowledge and practicing SDF with the type of specialty showed that pediatric dentists had statically significant (P<0.05) higher mean of knowledge 66.94 (SD=17.64) and practicing of SDF 61.93 (22.12) as compared to other specialties, while no statistically significant differences were found among the groups in the overall knowledge and practices based on years of experience and workplace. Attitudes were correlated with the three above variables (specialty, years of experience, and place of work) and showed that pediatric dentists had the highest satisfaction with the results of SDF (85.3%) and were more willing to recommend using it to others (91.2%) as compared to the other groups(P<0.05). No statistically significant differences were found with respect to the later variables.

Conclusion

The findings of this study showed that irrespective of the positive attitude of participants towards SDF, pediatric dentists were found to have higher knowledge and use SDF more than other dentists in different specialties. Furthermore, they showed higher satisfaction with its use. Years of experience and workplace had no effect on the level of knowledge or practice among participants.

## Introduction

Caries has become a global non-communicable microbial oral disease affecting various ages, from childhood to older population. It is considered to be a multifactorial disease caused by a combination of bacteria, defective host response, fermentable carbohydrates, and other modifying factors related to the host's lifestyle and affects the tooth's hard tissues [1,2]. Nearly 60%-90% of children and about 100% of adults have dental cavities worldwide [3]. According to a systematic review, dental caries were found to have a high negative impact on the oral health of children, adolescents, adults, and the elderly in Saudi Arabia [4]. Regular dental appointments are necessary to maintain good oral health and prevent dental diseases [5]. However, different factors may prevent people from attending or cause delayed dental appointments such as long waiting lists, cost, lack of time, and fear of dentists [4,5]. Other reasons that may complicate dental treatments are people with disabilities or behavioral difficulties [6,7]. Delaying dental treatment may lead to more serious irreversible diseases, from having only simple dental caries to more advanced problems, which may lead to the tooth being non-treatable and being extracted [8,9]. Furthermore, the effect has been reported to have a negative impact on oral health-related quality of life for the child and family, such as children having trouble sleeping (40%), eating (55.5%), and smiling (27.3%) due to poor status of their teeth. Moreover, a previous study found that half of the investigated parents reported that their kids were suffering from toothaches [10].

To overcome the consequences of dental caries, dentistry has made advancements toward more conservative procedures and techniques for the sake of saving teeth as much as possible [11]. Various preventive modalities have been introduced in dentistry aiming to limit the spread of caries among adults and children such as fluoride application and pit and fissure sealants [12]. Interventional methods to deal with caries have always been a chief concern for researchers and clinicians toward the preservation of tooth structure rather than the old concept of extension for prevention [13]. Routine dental procedures nowadays include restorative dentistry, atraumatic restorative treatment (ART), resin infiltration, and other techniques available [11].

One of the recent preventive techniques that has been introduced is the application of silver diamine fluoride (SDF). SDF was first discovered in Japan by Mizuho Nishino [14]. SDF is a colorless liquid that contains the sum of the antimicrobial effects of silver and the remineralizing action of fluoride [15], with a concentration of 38%, it is used to arrest dental caries in both primary and permanent dentitions. It is commonly used in children with behavioral difficulties and special needs, without the need for removal of sound tooth structure. Additionally, it is used as a desensitizing agent [16]. It is an efficient, effective, and inexpensive agent [17]. It can also be used as an alternative to restorative treatment in case of pandemics as what had been advised in COVID-19 to minimize the spread of aerosols [18,19].

A study was conducted using a questionnaire that was sent to members of the American Academy of Pediatric Dentistry (AAPD). The results revealed that only 3% of the participants had well or very well knowledge of SDF, and this knowledge was gained from dental schools, and during their residency. The results suggested that more education about SDF usage is required among the study sample [20]. Another study was conducted at Dammam, Saudi Arabia, the results showed adequate knowledge of SDF among dental specialists and postgraduate residents, but the knowledge was inadequate among general dentists, interns, and students. The study suggested that more academic education and clinical training are needed at all levels [21]. A qualitative study was conducted in Scotland, United Kingdom, assessing the knowledge and experience of using SDF in managing carious lesions in primary teeth. The results showed that participants were aware of the use of SDF; however, some participants indicated the presence of some barriers toward its use, including the black staining or the belief of it being toxic as perceived by the parents. Dental practitioners, however, acknowledged the valuable addition of SDF to pediatric dentistry treatment methods [22].

SDF was approved by the American Food and Drug Administration (FDA) in August 2014, and recently by the Saudi FDA in January 2020 [14] as a new preventive modality in treating dental caries. Because of the shortage of research conducted in Saudi Arabia regarding dentists' knowledge, practice, and attitudes toward SDF, establishing fundamental data for this relatively new yet important dental material will aid in enhancing the delivery of oral healthcare services pertaining to dental caries in the country, so, this study aimed to investigate the level of knowledge, attitudes, and practices about the use of SDF among dentists in Riyadh, Saudi Arabia.

## Materials and methods

Ethical approval

The study was approved by the College of Dentistry Research Center (CDRC NO.IR 0423) and the Institutional Review Board at University Hospital (IRB No E-22-7027), King Saud University.

This was a cross-sectional study taking place in different private and governmental hospitals, clinics, and dental colleges in Riyadh City, Saudi Arabia. The study period was from September 1, 2022 to March 19, 2023. The questionnaire was self-administered and some questions were taken from previously validated and published research [14,21,23]. To ensure the validity and reliability of the questionnaire items, a pilot survey was conducted by distributing a questionnaire to 10 dentists from various dental specialties, and their suggestions were taken into consideration. Additionally, Cronbach’s Alpha Coefficient test for reliability was found greater than 0.7 which indicates very good relatability.

Subsequently, an invitation was sent through WhatsApp and/ or email containing a web link and consent statement about the purpose of the research to 500 Saudi Commission for Health Specialists (SCFHS) registered dentists. The participants were assured that their participation was completely anonymous and confidential.

The sampling technique used for participant recruitment was performed by using a computerized simple random sample technique by selecting dentists from the SCFHS mailing list who fit our sample criteria and their communication numbers were available. Our target population was general practitioners (GP) (including general dentists and dental interns), pediatric dentists, public health practitioners, family dentists, Advanced Education in General Dentistry (AGED), and specialists in restorative dentistry.

The questionnaire was converted to an electronic form using Google Forms (Google Forms, 2019; a free web-based survey generator). It was written in English and comprised of 28 closed-ended questions with the following sections: Demographic data of participants included sex, nationality, type of specialty, current position or job title, years of experience, place of practice, and number of children they treat weekly. Three general questions about their perceived general knowledge about the uses of silver diamine fluoride (SDF) in dentistry, source of information, and if SDF is approved by the Saudi FDA to be used in dentistry. Knowledge/awareness of dentists towards SDF was assessed through eight questions: if they know what the SDF is, the correct concentration of SDF that is used in dentistry, its indications, contraindications, type of patients that can benefit from this treatment, level of safety, if they consider it a definitive treatment. and lastly, if it is possible to restore the treated tooth with composite restoration. Practice and efficacy of SDF experienced by the participants were explored through seven questions: Their familiarity with the clinical application of SDF on teeth, how often they use SDF in their practice, whether SDF is used to treat primary or permanent teeth or both, type of dental parts that SDF can treat (enamel, dentin, both), could they apply SDF if the dental caries is close to the pulp, the time interval that they apply SDF to the same tooth, what they do before application of SDF (local anesthesia, isolation, preparation of the tooth, none, do not know). The attitude of dentists was assessed by asking two questions: their satisfaction with the results of treatment with SDF and whether would they recommend others to use it for treating teeth.

Inclusion criteria

Female and male dentists (interns, general dentists, pediatric dentists, public health practitioners, family dentists, and restorative dentistry specialists), dentists who are working in private and governmental hospitals, clinics, and dental colleges in Riyadh, Saudi Arabia, and Saudi dentists were included.

Exclusion criteria

Dental undergraduates and non-Saudi dentists were excluded.

Sample size determination

The sample size was calculated using Power Analysis (G*) and Sample Size SPSS software version 25 (IBM Corp., Armonk, NY, USA). At alpha 0.05 with an effect size of 0.3 and power of 0.95, the total sample size was determined to be at least 220.

Statistical analysis

All responses were collected, edited, coded, and exported into spreadsheet SPSS for statistical analysis. Data were analyzed using frequency, percentage, mean, and standard deviation. Hypothesis testing was analyzed using the one-way ANOVA to determine the comparison of participants’ knowledge and Practice with three variables (participants’ specialty, place of work, and years of experience), while Chi-square was used for comparison of respondent’s attitude about the use of SDF and the above same variables. The level of significance of alpha was set at 0.05, so any test with a P-value less than 0.05 was considered significant.

## Results

Out of 500 recipients of the survey link, a total of 275 responses were received from dentists working in hospitals, colleges, and private practice in Riyadh City, Saudi Arabia, of which 10 were excluded because they were not Saudis ending with 265 responses suitable for analyses giving a response rate of 53%. The demographic background is presented in Table 1. Out of 265 dentists, 43% were males and 57% were females. The majority of the respondents were from nonacademic sectors (hospitals and private clinics), and about half of them (53.9%) were treating one to five children weekly while around one-fifth of them (20.8%) were treating more than 10 children per week. Regarding the type of specialty, approximately half of the respondents were general dentists or dental interns which were combined in the table under GP, followed by pediatric dentists (Pedo) (25%), then restorative dentistry (Resto) (20.0%) which also include dentists from family dentistry, AGED and public health specialty that were combined due to small number of respondents from that specialty. Almost half of the participating dentists (55.5%) believed that they were very familiar with the uses of SDF in dentistry while 44.5% thought that they had very little knowledge about it. Regarding dentists’ knowledge of the use of SDF has been approved by the Saudi FDA, more than half of the participants (51.7%) do not know that it was approved.

**Table 1:** Characteristics of Saudi dentists participating in the study *Includes general dentists and dental interns **Includes family, Advanced Education in General Dentistry (AGED) and public health

Item	Number (%)
Sex	
Male	114(43)
Female	151(57)
Workplace	
Academic	76(28.7)
Nonacademic	143(53.9)
combination	46(17.4)
Number of treating children	
0	38(14.3)
1 - 5	146(55.1)
6 - 10	26(9.8)
>10	55(20.8)
Years of experience	
0-1	90(34.0)
2 -5	115(43.4)
=>6	60(22.6)
Specialty type	
General practitioner*	144(54.3)
Pediatric dentist	68(25.7)
Restorative dentistry**	53(20.0)
Awareness of uses of SDF in dentistry	
Very aware	147(55.5)
Little aware	118(44.5)
Do You know if SDF was approved in Saudi Arabia?	
Yes	128(48.3)
No	137(51.7)

**Figure 1:**
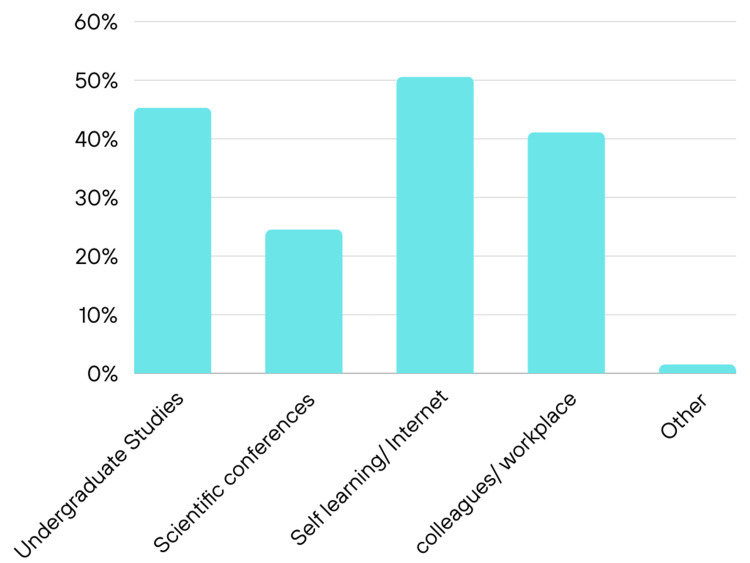
Source of information about SDF among participants SDF - silver diamine fluoride

Figure 1 illustrates the source of information about SDF. Most of the participants acquired their knowledge through self-learning/internet (50.6.%) followed by basic education during undergraduate studies (45.3%).

Overall mean score knowledge and practice among dentists

Participants' overall mean score knowledge and practice about SDF was computed by getting the total correct score of each dentist and divided by the number of corrected answers in the questionnaire. The mean knowledge (based on eight questions in the questionnaire) is 49.13 (SD=19.81) in which the highest correct score that participants achieved was 92 while the minimum score was 7.10 out of 100. Regarding using SDF (seven questions), the mean score of practice is 43 (SD=26.12), in which the highest answer was 93.30 and the lowest score was zero out of 100.

Table 2 correlates the mean score of knowledge and practice with the type of specialty. It showed a higher mean of knowledge 66.94 (SD=17.64) among pediatric dentists as compared with GP and restorative dentistry specialists and the differences were statistically significant (P=0.001).

**Table 2:** Percentage of total score-knowledge, practice with group specialty *Includes general dentists and dental interns ** includes family, AGED (Advanced Education in General Dentistry) and public health ***ANOVA Test

	N	Mean (SD)	95% confidence interval Lower	Upper	P-value***
%-score-Knowledge					
GP*	144	53.80(23.67)	49.90	57.7	
Pedo	68	66.94(17.64)	62.67	71.22	0.001
Resto**	53	54.58(22.46)	48.39	60.77	
Total	265	57.33(22.67)	54.59	60.07	
%-score-Practice					
GP*	144	48.33(26.03)	44.04	52.62	
Pedo	68	61.93(22.23)	56.55	67.31	0.001
Resto**	53	41.97(25.11)	35.05	48.89	
Total	265	50.55(25.85)	47.42	53.67	

The table presents the mean score of practice of participants as correlated by their specialty. The mean practice of SDF among pediatric dentists was found higher than that of other groups 61.93 (22.23) and this difference was statistically significant (P=0.001).

Knowledge and practice with participants’ years of experience

Table 3 presents the correlation of participants' scores of knowledge and practice with years of experience. There was a slight increase in overall knowledge about SDF with the increase in work experience but there was no statistically significant difference among the groups(P>0.05). Similarly, the same table shows a slight increase in the mean of practice with the increase of years of experience, but this was not found to be statistically significant (P>0.05).

**Table 3:** Percentage of total score-knowledge, practice with participants’ years of experience *ANOVA Test

Item	N	Mean (SD)	95%confidence Interval Lower	upper	P-value*
%-score-Knowledge					
0-1 yr.	90	53.97(24.49)	48.85	59.10	
2-5 Yr.	115	58.65(23.29)	54.35	62.95	0.23
=>6	60	59.84(17.91)	55.21	64.47	
Total	265	57.33(22.67)	54.59	60.07	
%-score-practice					
0-1 yr.	90	48.07(25.61)	42.71	53.43	
2-5 yr.	115	50.33(26.17)	45.49	55.16	0.30
=>6 yr.	60	54.69(25.43)	48.12	61.26	
Total	265	50.55(25.83)	47.42	53.67	

Knowledge and practice with participants’ place of work

Table 4 shows the correlation between participants' scores of knowledge and place of work, there was no difference in the overall knowledge about SDF among academic, nonacademic, or dentists who work in both academic and nonacademic sectors (p>0.05). Similarly, the same table exhibits that there was no statistically significant difference in practicing SDF and different workplaces of dentists (P>0.05).

**Table 4:** Percentage of total score knowledge and practice with participants' place of work *ANOVA test

Item	N	Mean (SD)	95%confidence Interval Lower	Upper	P-value*
%-score-Knowledge					
Academic	76	54.40(23.34)	49.04	59.71	
Non-Academic	143	56.84(22.45)	53.13	60.56	0.08
Both	46	63.72(21.44)	57.36	70.09	
Total	265	57.33(22.67)	54.59	60.07	
%-score-practice					
Academic	76	47.52(25.87)	41.61	53.43	
Non-Academic	143	49.71(25.59)	45.48	53.94	0.07
Both	46	58.16(25.64)	50.54	65.77	
Total	265	50.55(25.84)	47.42	53.67	

Participants’ attitude toward the use of SDF

Table 5 shows that pediatric dentists had higher satisfaction with the results of SDF 58 (85.3%) and they are more willing to recommend using it (91.2%) than other specialties (GP and restorative dentistry) and this difference was found to be statistically significant (P=0.0001).

**Table 5:** Participants’ attitude toward the use of SDF by their specialty *Includes general dentists and dental interns **Includes family, Advanced Education in General Dentistry (AGED) and public health ***Chi-square SDF - silver diamine fluoride

	GP*	Pedo	Resto**	Total	P-value***
Are you satisfied with results of SDF					
No no(%)	62(43.1)	10(14.7)	15(28.3)	87(32.8)	
Yes no(%)	82(56.9)	58(85.3)	38(71.7)	178(67.2)	0.0001
Total no(%)	144(100)	68(100)	53(100)	265(100)	
Would you recommend SDF to others					
No no(%)	53(36.8)	6(8.8)	13(24.5)	72(27.2)	
Yes no(%)	91(63.2)	62(91.2)	40(75.5)	193(72.8)	0.0001
Total no(%)	144(100)	68(100)	53(100)	265(100)	

Attitudes toward the use of SDF with participants’ years of experience

Table 6 presents the correlation between the satisfaction level of participants and years of experience. It shows that dentists with two to five years of experience were more satisfied (73.9%) than the other groups, but this difference was not statistically significant (P>0.05).

**Table 6:** Participants’ attitude toward the use of SDF by the year of experience *Chi-square SDF - silver diamine fluoride

Questions	0-1 Yr.	2-5 Yr.	=>6	Total	P-value*
Are you satisfied with results of SDF					
No no (%)	36(40.0)	30(26.1)	21(35.0)	87(32.2)	
Yes no (%)	54(60.0)	85(73.9)	39(65.0)	178(67.2)	0.1
Total no (%)	90(100)	115(100)	60(100)	265(100)	
Would you recommend SDF to others					
No no(%)	27(30.0)	28(24.3)	17(28.3)	72(27.2)	
Yes no(%)	36(70.0)	87(75.7)	43(71.7)	193(72.8)	0.65
Total no (%)	90(100)	115(100)	60(100)	265(100)	

Table 7 shows participants’ level of satisfaction with the use of SDF in participants’ workplaces. No statistically significant correlation was found in relation to different workplaces (P>0.05).

**Table 7:** Attitudes toward the use of SDF by participants’ place of work *Chi-square SDF - silver diamine fluoride

Questions	Academic	Non-Academic	Both	Total	P-value*
Are you satisfied with results of SDF					
No no (%)	25(32.9)	47(32.9)	15(32.6)	87(32.8)	
Yes no (%)	51(67.1)	96(67.1)	31(67.4)	178(67.2)	0.99
Total no (%)	76(100)	143(100)	46(100)	265(100)	
Would you recommend SDF to others					
No no (%)	19(25.0)	40(28.0)	13(38.3)	72(27.2)	
Yes no (%)	57(75.0)	103(72.0)	33(71.7)	193(72.8)	0.88
Total no (%)	76(100)	143(100)	46(100)	265(100)	
Questions	Academic	Non-Academic	Both	Total	P-value*
Are you satisfied with results of SDF					
No no (%)	25(32.9)	47(32.9)	15(32.6)	87(32.8)	
Yes no (%)	51(67.1)	96(67.1)	31(67.4)	178(67.2)	0.99
Total no (%)	76(100)	143(100)	46(100)	265(100)	
Would you recommend SDF to others					
No no (%)	19(25.0)	40(28.0)	13(38.3)	72(27.2)	
Yes no (%)	57(75.0)	103(72.0)	33(71.7)	193(72.8)	0.88
Total no (%)	76(100)	143(100)	46(100)	265(100)	

## Discussion

Recently, in 2020, the Saudi FDA approved using SDF as a preventive measure in dentistry, so the purpose of this study is to assess the knowledge, attitudes, practices, and educational background among Saudi clinicians who are using SDF material in Riyadh, Saudi Arabia.

The study showed that more than 50% of participants did not know about the recent approval of SDF by the Saudi FDA in dentistry which may indicate the presence of some communication obstacles among the community of dentistry. Almost the same percentage believe that they were very aware of the use of this product in dentistry. They acknowledged that they obtained their information from self-learning/ internet and in their workplace, as well as from their undergraduate studies which means that even though SDF was not approved by the Saudi FDA, the dental colleges kept students updated about different modalities of dental treatments and included it in their dental curricula at universities. The participants showed encouraging behaviors by updating their professional information through several methods, similar results were reported by other studies [20,21,23].

Regarding knowledge of SDF, the results of the current study showed that the total knowledge score of participants is 49.13 (SD=19.81) which means that approximately half of the investigated dentists had enough knowledge. Correlation of the mean Knowledge score and different specialties showed that pediatric dentists had the highest mean 61.93 (SD=21.22) as compared to other specialties, this may be because SDF is mainly beneficial as a treatment alternative for very young, uncooperative children, or those with medical or mental disabilities, these results were in accordance with other studies [20,23-25]; however, this finding is contrary to that reported by Alajlan et al.'s study [14] as the restorative specialty was observed to have a higher mean of knowledge toward SDF compared to those in pediatric dentistry, this was possibly noted because of the distribution of sample size for different specialties in both studies, while in Schroe et al.'s study, they did not find any statistical difference in knowledge between pediatric and general dentists in which they attributed to the recent approval of SDF in the Netherlands [25].

With respect to the use of SDF in participants’ practice, findings showed less than half of participants are using this product in their practice, this is probably because about one-quarter of the sampled dentists are fresh graduates, so they did not have a chance to use this material in their clinics considering its recent approval in Saudi Arabia. When using SDF was compared among dentists in different specialties, a statistically higher percentage of pediatric dentists was found to use this material compared to other dentists, our result is consistent with Schroe et al.'s study [25].

In regard to the participant's attitude toward the use of SDF in their clinics, the results showed that higher percentage of pediatric dentists (85.3%) were satisfied with the outcome of SDF compared to other dentists, consequently, 91.2% of them would recommend its use to others, pediatric dentists who showed more knowledge about SDF were found more likely to have positive attitude and use it more frequently than others indicating that further education about SDF may help increase its use by dental practitioner. Our results were in accordance with other studies [21,25], while disagreeing with the findings of other studies conducted in Saudi Arabia [14] which may be attributed to the different sample sizes and dental specialties that were included in the two studies.

Considering the comparison of knowledge, practice, and attitude among different specialties according to years of experience and place of work, no statistical differences were detected among groups, this may be due to the unavailability of this material in their place of work. 

The present study encountered some limitations; low response rate since the research was based on an online survey. Consequently, we end up with a relatively small number of study sample size. Another possible cause of the low response rate could be that the non-responders were unfamiliar with SDF, which led to non-participation. An additional limitation was differences and dissimilarity in the number of responses among different groups of specialties which led to combining two or three similar specialties which may contribute to differences in the overall results. 

Future research should therefore clarify other aspects that we could not explore in this research including satisfaction with the amount of educational materials available, obstacles to getting sufficient knowledge about new dental information, and include larger number of responses from different specialties. Regardless of the above-mentioned limitations, this research gives an insight into the current participants’ knowledge and level of practicing SDF in their workplace. These insights are crucial for improving dental healthcare strategies. Furthermore, the current results necessitate the need for more effective communication among the profession of dentistry, in addition to the importance of providing dentists with the latest information in dentistry.

## Conclusions

The study participants exhibited positive attitudes toward SDF and were nearly less than average in the mean knowledge and practice scores. Furthermore, the study revealed that pediatric dentists exhibited the highest scores in knowledge and practice of SDF among other specialties and were the most satisfied with it. No effect of years of experience and place of work on the level of knowledge or practice of SDF.
